# Centipede Polypeptide Affects the Inflammatory Reaction and Ferroptosis of Liver Cancer Cells Through the p53/TRAIL Pathway

**DOI:** 10.1111/jcmm.70844

**Published:** 2025-10-13

**Authors:** Xingru Xing, Linzhu Lu, Zhen Huang, Jiawei Wang, Lihuai Wang, Yuxing Hu, Shan Yin, Sha Tian

**Affiliations:** ^1^ Department of Internal Medicine College of Integrated Chinese and Western Medicine of Hunan University of Chinese Medicine Changsha Hunan China; ^2^ The First Hospital of Hunan University of Chinese Medicine Changsha Hunan China; ^3^ State Key Laboratory of Quality Research in Chinese Medicine Macau University of Science and Technology Taipa Macao China

**Keywords:** centipede polypeptide, ferroptosis, inflammatory reaction, liver cancer, p53/TRAIL pathway

## Abstract

Various extracts from centipedes have therapeutic effects on liver cancer. This study aims to illustrate the impact and mechanism of centipede polypeptide (CP) on liver cancer. The suitable CP concentration and liver cancer cells were screened through CCK‐8 analysis. The expression of proteins was analysed by Western blot. The level of cytokines and markers was measured by ELISA and biochemical kits. The apoptosis rate of cells was analysed by TUNEL staining and flow cytometry. Histopathological changes were observed by HE staining. The expression of Ki‐67 and caspase‐3 was assessed by IHC staining. The combination of CP and p53 was simulated by molecular docking. 200 μg/mL CP and HepG2 cells were applied in experiments. CP significantly upregulated p53, TNF‐associated apoptosis‐inducing ligand (TRAIL), TRADD and TRAF expression in HepG2 cells and tumour tissues (*p* < 0.05), suggesting p53‐dependent activation of the TRAIL pathway. CP raised the levels of IL‐6, IL‐1β, TNF‐α, MDA and ROS, and decreased those of IL‐10, TGF‐β1 and SOD in HepG2 cell supernatant and serum of nude mice (*p* < 0.05). CP promoted cell apoptosis and reduced the levels of ALT and AST to inhibit the progression of cancer. Molecular docking showed that CP could bind stably to p53 (*p* < 0.05). Silencing of p53 restrained the activation of the p53/TRAIL pathway and reduced the level of inflammatory reaction and ferroptosis, thus reversing the therapeutic effect of CP on liver cancer (*p* < 0.05). CP affected the inflammatory reaction and ferroptosis of liver cancer cells through the p53/TRAIL pathway.

**Abbreviations**
3D: three‐dimensional
ALT: alanine transaminase
AST: aspartate transaminase
CAS: Chemical Abstracts Service
CCK‐8: cell counting kit‐8
CP: centipede polypeptide
ELISA: enzyme‐linked immunosorbent assay
GPX4: glutathione peroxidase 4
GSH: glutathione
HE: haematoxylin–eosin
HRP: horseradish peroxidase
IHC: immunohistochemistry
IL‐6: interleukin‐6
MDA: malondialdehyde
NC: nitrocellulose
PCa: prostate cancer
PTGS2: prostaglandin‐endoperoxide synthase 2
ROS: radical oxygen species
SLC7A11: solute carrier family 7 member 11
SOD: superoxide dismutase
TCM: traditional Chinese medicine
TGF‐β1: transforming growth factor‐β1
TNF‐α: tumour necrosis factor‐α
TRADD: TNF‐R1‐associated death domain
TRAF: TNF receptor‐associated factor
TRAIL: TNF‐associated apoptosis‐inducing ligand

## 1 Introduction

Liver cancer, classified as a malignant tumour, occurs in the liver and is characterised by high incidence and aggressiveness [1]. Despite advances in research and the development of treatment methods, liver cancer is still one of the most common causes of cancer‐related deaths worldwide. Nowadays, surgery [2], radiotherapy [3], chemotherapy [4] and immunotherapy [5] are the main therapy methods for liver cancer. Surgery is the standard treatment for patients with early liver cancer, but its recurrence rate is high [6]. For patients with advanced liver cancer, the kinase inhibitor is a better choice, but it is easy to develop drug resistance and certain toxicity in long‐term use [7]. As a result of the insidious occurrence and development, most patients are found in the middle and late stages. Thus, it is essential to seek a safe and effective alternative medicine to prevent and treat liver cancer.

Traditional Chinese medicine (TCM) can reduce toxicity, enhance effectiveness, improve immune function and prolong survival [8]. Centipedes, one of the TCM materials, whose chemical composition mainly includes enzymes, peptides, polysaccharides, fatty acids, amino acids, trace elements, and so on [9, 10]. The pharmacological effects of the centipede mainly include antitumour, protection of the cardiovascular and cerebrovascular system, analgesia, promotion of digestion, regulation of immune function, and so on [11]. In recent years, the inhibitory effects of the centipede oligopeptides and extracts on tumour cells through apoptosis‐related pathways have attracted increasing attention [12, 13]. The research has verified the potential of Centipede Scolopendra Extract in treating liver cancer [14]. In our previous research, Scolopentide was extracted, purified and identified from *Scolopendra subspinipes mutillans*, and its activity in inducing apoptosis of liver cancer cells was preliminarily verified by activating TNF‐related apoptosis‐inducing ligand (TRAIL) receptors (DR4 and DR5) [15]. However, the regulatory mechanism of liver cancer is very complex, and the specific mechanism of centipede peptides (CP) from *Scolopendra subspinipes mutillans* regulating liver cancer still needs further clarification.

Currently, increasing attention has been paid to the relationship between tumours and inflammatory reactions. In the process of tumorigenesis, inflammatory cells and inflammatory cytokines participate in forming the inflammatory microenvironment of tumorigenesis and the process of tumour invasion and metastasis [16]. In addition, ferroptosis depends on iron and radical oxygen species (ROS) and is associated with a variety of diseases, especially liver diseases [17]. Studies have shown that activation of intracellular p53 can promote the expression of TRAIL; targeting p53 may be an important strategy for overcoming TRAIL resistance in cancer therapy [18, 19]. Under the combined treatment of ferroptosis inducers and TRAIL, the p53‐independent CHOP/DR5 axis is involved in the synergistic enhancement of apoptosis in human colon cancer cells [20]. In addition, inhibiting TRAIL‐induced apoptosis can alleviate cell inflammation levels [21]. Studies have shown that induction of autophagy and upregulation of p53 expression can promote apoptosis of liver cancer cells [22, 23]. For example, allicin has promoted apoptosis of liver cancer cells by regulating apoptosis and autophagy through the p53 gene [24]. TNF‐associated apoptosis‐inducing ligand (TRAIL) is a potential anticancer agent in several cancers, selectively inducing apoptosis of cancer cells without damaging normal cells [25]. However, the resistance of cancer cells to TRAIL limits its application [26]. Therefore, the development of drugs that activate the TRAIL pathway is critical for the treatment of liver cancer [27]. Nutlin‐3 promotes TRAIL‐induced apoptosis of liver cancer cells by activating p53 [28]. Betulinic acid promotes TRAIL to inhibit tumour progression by activating the p53/caspase‐3 pathway [29]. Adiponectin regulates the p53/TRAIL/caspase‐8 pathway in the treatment of thioacetamide‐induced liver cancer [30]. Cantharidin induces apoptosis of human Hep3B cells through the p53‐independent pathway of TRAIL/DR5 signal transduction [31]. Therefore, activation of the p53/TRAIL pathway is a potential strategy for the treatment of liver cancer.

With the p53/TRAIL pathway as the direction, the therapeutic effect and mechanism of CP on liver cancer were explored to benefit the development of new methods for liver cancer treatment.

## 2 Materials and Methods

### 2.1 Cell Culture

Normal human liver cells WRL68 (BNCC353696, BNCC, China) were kept in Roswell Park Memorial Institute (RPMI)‐1640 medium while liver cancer cells HepG2 (BNCC338070, BNCC, China), Huh‐7 (BNCC337690, BNCC, China) and HCCLM3 (BNCC342335, BNCC, China) cells were kept in Dulbecco's modified Eagle medium (DMEM), containing 10% fetal bovine serum (FBS) (10099141, Gibco, USA) and 1% Penicillin/Streptomycin (SV30010, Beyotime, China), and cultured in a humidified incubator (DH‐160I, SANTN, China) under 5% CO_2_ at 37°C [32]. They were used in experiments when the confluence reached 70%–80%.

### 2.2 Cell Grouping and Treatment

**FIGURE 1:**
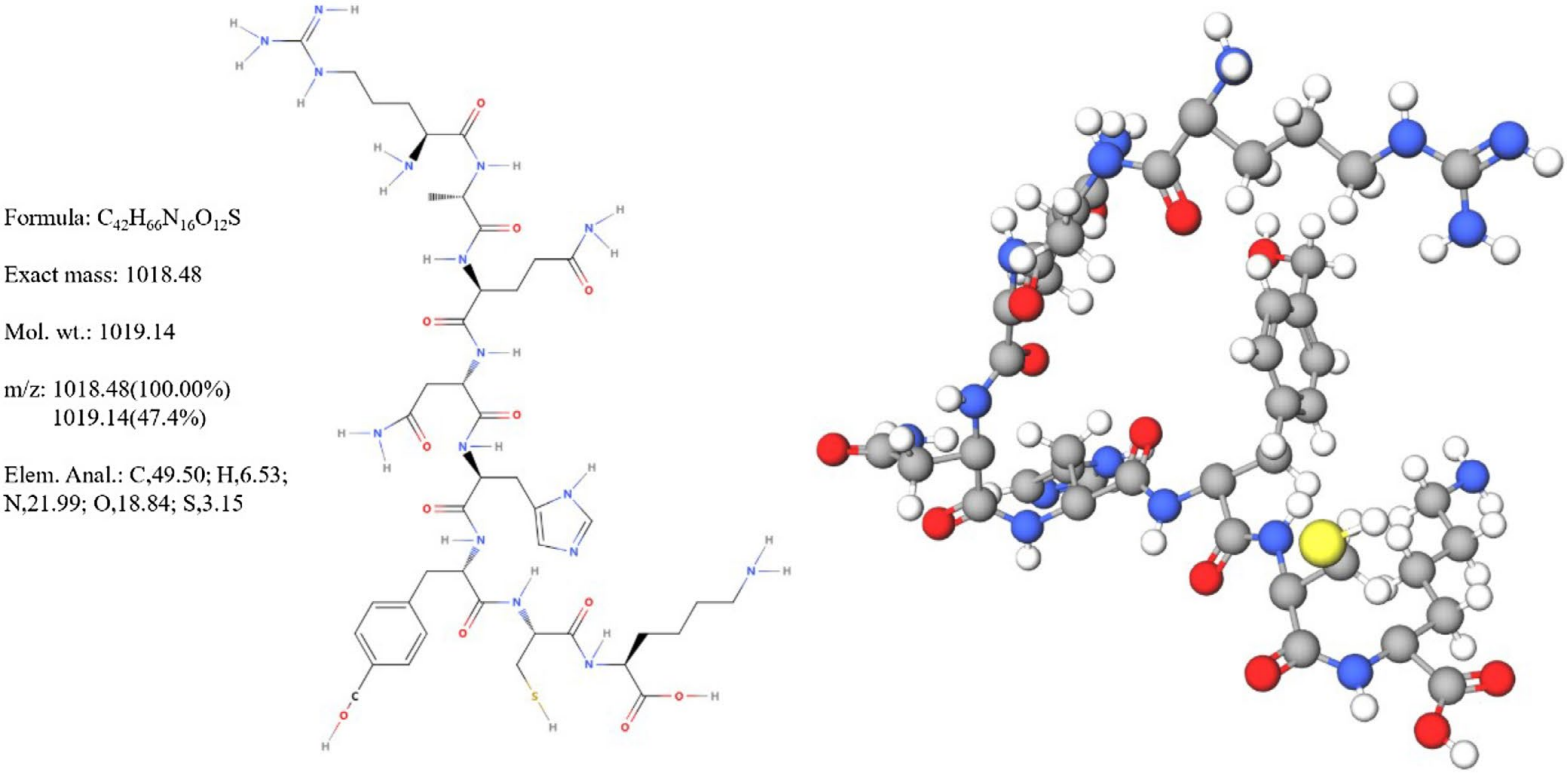
Chemical structure diagram of CP.

CP was extracted from *Scolopendra subspinipes mutilans* using the enzymatic hydrolysis and acetone precipitation method [15]. The chemical structure diagram of CP was displayed in Figure 1. In the experiment to screen CP concentration and liver cancer cells, WRL68, HepG2, Huh‐7 and HCCLM3 cells were treated with CP (0, 50, 100, 150 and 200 μg/mL) for 0, 24 and 48 h, respectively [15]. Cell grouping 1: Control, 50 μg/mL CP, 100 μg/mL CP, 150 μg/mL CP and 200 μg/mL CP. HepG2 cells in the control group were cultured under normal conditions, while cells in the treatment groups were exposed to CP at the corresponding concentrations for 48 h. Cell grouping 2: Control, CP, CP+si‐NC and CP+si‐p53. These groupings were designed to explore the regulatory role of p53 in CP's effects on inflammation and ferroptosis in liver cancer. HepG2 cells in the Control group were cultured normally. HepG2 cells in the CP group were treated with 200 μg/mL CP for 48 h. HepG2 cells in the CP+si‐NC group were transfected with si‐NC plasmids (5′‐UUCUCCGAACGUGUCACGU‐3′), and treated with 200 μg/mL CP for 48 h. HepG2 cells in the CP+si‐p53 group were transfected with si‐p53 plasmids (5′‐UCAAAUCAUCCAUUGCUUG‐3′, HG‐Si007157, Abiowell, China), and treated with 200 μg/mL CP for 48 h.

### 2.3 Cell Counting Kit‐8 (CCK‐8) Assay

After being digested by trypsin (AWC0232, Abiowell, China), WRL68, HepG2, Huh‐7 and HCCLM3 cells (1 × 10^4^ cells/well) were cultured at 37°C. After cell adhesion, cells in each group were treated with the corresponding drug for 0, 24 and 48 h. 100 μL of medium containing 10% CCK‐8 was added to replace the drug‐containing medium. Cells were cultured at 37°C for 4 h and analysed with a multifunctional microplate reader at 450 nm (MB‐530, HUISONG, China) [33].

### 2.4 Western Blot

Different groups of cells or tissues were mixed with RIPA lysate (AWB0136, Abiowell, China) to extract total proteins. The proteins were transferred to nitrocellulose (NC) membranes after SDS‐PAGE. After being blocked in 5% skimmed milk (AWB0004, Abiowell, China) for 1.5 h, they were incubated with the primary antibody at 4°C overnight. Primary antibodies included: p53 (1:5000, 10442‐1‐AP, Proteintech, USA), TRAIL (1 μg/mL, ab42121, Abcam, UK), TNF‐R1‐associated death domain (TRADD, 1:1000, 15468‐1‐AP, Proteintech, USA), TNF receptor‐associated factor (TRAF, 1:1000, ab300075, Abcam, UK), glutathione peroxidase 4 (GPX4, 1:1000, 67763‐1‐Ig, Proteintech, USA), solute carrier family 7 member 11 (SLC7A11, 1:5000, ab175186, Abcam, UK), prostaglandin‐endoperoxide synthase 2 (PTGS2, 1:3000, ab179800, Abcam, UK) and β‐actin (1:5000, 66009‐1‐Ig, Proteintech, USA). The membranes were incubated with HRP‐labelled secondary antibodies for 1.5 h. The membranes were incubated with ECL reagent (AWB0005, Abiowell, China) and followed by imaging. The expression levels of proteins were analysed by Quantity One 4.6.6 with β‐actin as the reference protein.

### 2.5 Molecular Docking

The three‐dimensional (3D) structure diagram of p53 was searched from the PDB database (https://www.rcsb.org/). After the removal of water molecules by PyMOL (ver.2.3.1), the addition of hydrogen atoms by Autodock tools (ver.1.5.6), calculation of the charge, and setting of the atomic type, it was saved in PDBQT format. The Chemical Abstracts Service (CAS) number of CP was searched from the PubChem database (https://pubchem.ncbi.nlm.nih.gov/) to download the 3D structure diagram. Its energy was minimised with Chem3D Pro and was saved in mol2 format. VINA 1.1.2 software was used for docking, and the results were visualised with Discovery Studio software. Finally, the 3D molecular docking model was output [34].

### 2.6 Nude Mouse Tumorigenicity Assay and Intervention

Female nude mice (4 weeks, 12–15 g) were brought from Hunan Slyke Jingda Laboratory Animal Co. Ltd. The experiments began after the nude mice received adaptive feeding for 1 week. In animal experiment 1, each nude mouse was subcutaneously inoculated with 5 × 10^6^ HepG2 cells into the right axilla [35]. After 14 days, tumour‐bearing nude mice were randomly divided into four groups: control, low‐dose CP (low‐CP), medium‐dose CP (mid‐CP) and high‐dose CP (high‐CP) groups. Nude mice in Low‐CP, Mid‐CP and High‐CP groups were intraperitoneally injected with 20 g/kg, 100 g/kg and 200 g/kg CP, respectively. This experiment was conducted to investigate the effects of CP on liver cancer. In animal experiment, to explore the role of p53 in liver cancer treatment by CP, nude mice were randomly divided into Control, CP, si‐NC+CP and si‐p53+CP groups (six mice/group). Cells were subcutaneously inoculated into the right axillary region of nude mice. The inoculated cells, corresponding to each group, were as follows: HepG2, HepG2, si‐NC‐transfected HepG2 and si‐p53‐transfected HepG2 (5 × 10^6^ cells per mouse). Nude mice in the CP, si‐NC+CP and si‐p53+CP groups were intraperitoneally injected with 200 g/kg CP. Nude mice in the Control groups in all experiments were intraperitoneally injected with the same dose of normal saline. The injection volume of CP in experiments was 100 μL/10 g once a day for 14 days. On day 29, nude mice were anaesthetised with sodium pentobarbital (50 mg/kg) to obtain whole blood [36]. They were then euthanised by intraperitoneal injection of sodium pentobarbital (150 mg/kg) [37]. The tumour tissue was harvested, photographed and then measured for volume and weight. The tumour tissues were fixed, embedded and sectioned for analysis of HE, IHC and TUNEL staining. The animal experiment was approved by the Hunan University of Chinese Medicine Committee (No. LLBH‐202206240002).

### 2.7 Enzyme‐Linked Immunosorbent Assay (ELISA) and Biochemical Detection

The whole blood of nude mice and HepG2 cell supernatant was centrifuged at 4°C at 1000 *g* for 15 min to obtain the supernatant for detection. The levels of interleukin‐6 (IL‐6, KE10007, Proteintech, USA), IL‐10 (KE10008, Proteintech, USA), IL‐1β (KE10003, Proteintech, USA), tumour necrosis factor‐α (TNF‐α, KE10002, Proteintech, USA) and transforming growth factor‐β1 (TGF‐β1, KE10005, Proteintech, USA) in HepG2 cell supernatant and serum of nude mice were measured by ELISA kits. The levels of malondialdehyde (MDA, A003‐1, NJJCBIO, China) in HepG2 cell supernatant and tumour tissues, superoxide dismutase (SOD, A001‐3, NJJCBIO, China) and ROS (F9689‐B, FANKEW, China) in HepG2 cell supernatant, glutathione (GSH, A006‐2‐1, NJJCBIO, China) and Fe^2+^ (ab83366, Abcam, UK) in HepG2 cells, alanine transaminase (ALT, C009‐2‐1, NJJCBIO, China) and aspartate transaminase (AST, C010‐2‐1, NJJCBIO, China) in serum of nude mice were assayed by biochemical kits.

### 2.8 Flow Cytometry

As for the detection of apoptosis rate, about 2 × 10^5^ HepG2 cells were suspended with 500 μL of Binding buffer. According to the instructions of the kits (KGA1030, KeyGen Biotech, China), the above solution was incubated with 5 μL of Annexin V‐APC and 5 μL of propidium iodide. Detection by flow cytometry was carried out after the reaction in the dark for 10 min.

### 2.9 Haematoxylin–Eosin (HE) Staining

The slices were dewaxed in xylene and dehydrated with gradient ethanol (75%–100%). After being stained with haematoxylin (AWI0001a, Abiowell, China) for 5 min and rinsed with distilled water, the slices were returned to blue with PBS. After being stained with eosin (AWI0029a, Abiowell, China) for 5 min, followed by dehydration with gradient ethanol (95%–100%). The slices were placed in xylene for 10 min for transparency and observed by a microscope after being sealed with neutral gum (AWI0238a, Abiowell, China).

### 2.10 Immunohistochemistry (IHC) Staining

The expressions of Ki‐67 and caspase‐3 were analysed by IHC staining. Each batch of experiments was set up simultaneously with the negative control and the positive control. The slices were dewaxed and dehydrated with gradient ethanol (75%–100%). After antigen repair, endogenous enzymes in tissues were inactivated by 1% periodic acid. The slices were incubated with the primary antibodies of Ki‐67 (1:300, ab16667, Abcam, UK) and caspase‐3 (1:100, ab32351, Abcam, UK) at 4°C overnight and 100 μL of horseradish peroxidase (HRP) goat anti‐rabbit IgG (1:100, AWS0005, Abiowell, China) at 37°C for 30 min. Then 100 μL of DAB (ZLI‐9017, ZSBG‐Bio, China) was added and incubated for 5 min. The slices were re‐stained with haematoxylin, placed in xylene for transparency, sealed with neutral gum and observed by a microscope (BA410‐T, Motic, China). Three fields were selected in each group, each with magnifications of 100 and 400. The immunostaining results were evaluated independently by two pathologists, ST and ZH. The software used for image analysis was Image‐Pro‐Plus (v6.0, MEDIA CYBERNETICS, USA). The positive rate was calculated to represent the expressions of Ki‐67 and caspase‐3, which equals the ratio of the number of positively stained nuclei to the total number of nuclei in 400‐fold fields of view [38, 39]. The negative control sample was the tumour tissues of the mice in this work, and the IHC staining detection process omitted the primary antibody but included all other steps. For the detection of Ki67 expression, the positive control sample was mouse spleen tissue. For the detection of caspase‐3 expression, the positive control sample was human endometrial tissues.

### 2.11 TUNEL Staining

TUNEL staining (40306ES50, Yeasen, China) was applied to assay the apoptosis rate of HepG2 cells in tumour tissues [40]. After being dewaxed in xylene and dehydrated with gradient ethanol (75%–100%), the slices were reacted with 100 μL of Proteinase K at 37°C for 20 min, followed by 100 μL of equilibration buffer at 25°C for 30 min. After being incubated with 50 μL of TDT at 37°C for 1 h, the slices were stained with DAPI at 37°C for 10 min, sealed with glycerol, and observed by a fluorescence microscope.

### 2.12 Statistical Analysis

Experimental data were processed by GraphPad Prism 8.0 and expressed as mean ± standard deviation. One‐way ANOVA and two‐way ANOVA were used for comparison between multiple groups. The difference was significant as *p* < 0.05 [41].

## 3 Results

### 3.1 Screening of CP Concentration and Liver Cancer Cells

**FIGURE 2:**
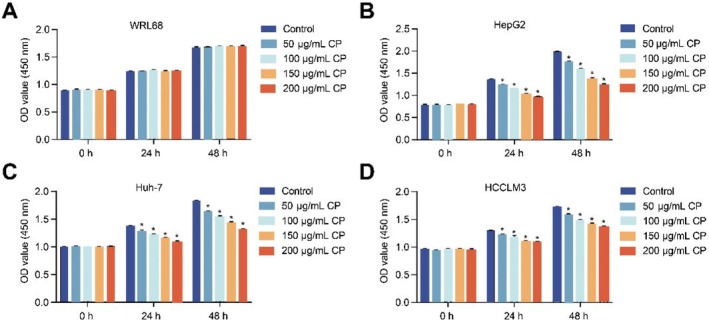
Screening of CP concentration and liver cancer cells. (A–D) The proliferation capacity of WRL68, HepG2, Huh‐7 and HCCLM3 cells was analysed by CCK‐8. The experiment was repeated three times. The data were presented as the mean ± SD. *p < 0.05 vs. control.

CCK‐8 assay was applied to screen a suitable concentration and liver cancer cells. The results displayed that different concentrations of CP did not affect the proliferation capacity of WRL68 cells. However, CP inhibited the proliferation of HepG2, Huh‐7 and HCCLM3 cells in a dose‐dependent manner, with 200 μg/mL CP having the strongest inhibitory effect. Moreover, the inhibition rates of HepG2, Huh‐7 and HCCLM3 at 48 h were calculated to be 37.56%, 27.75% and 20.34% respectively, and that of HepG2 cells was the highest (Figure 2A–D). In summary, 200 μg/mL CP and HepG2 cells were selected for subsequent experiments.

### 3.2 CP Affected the Inflammatory Reaction of Liver Cancer Cells by Regulating the p53/TRAIL Pathway

**FIGURE 3:**
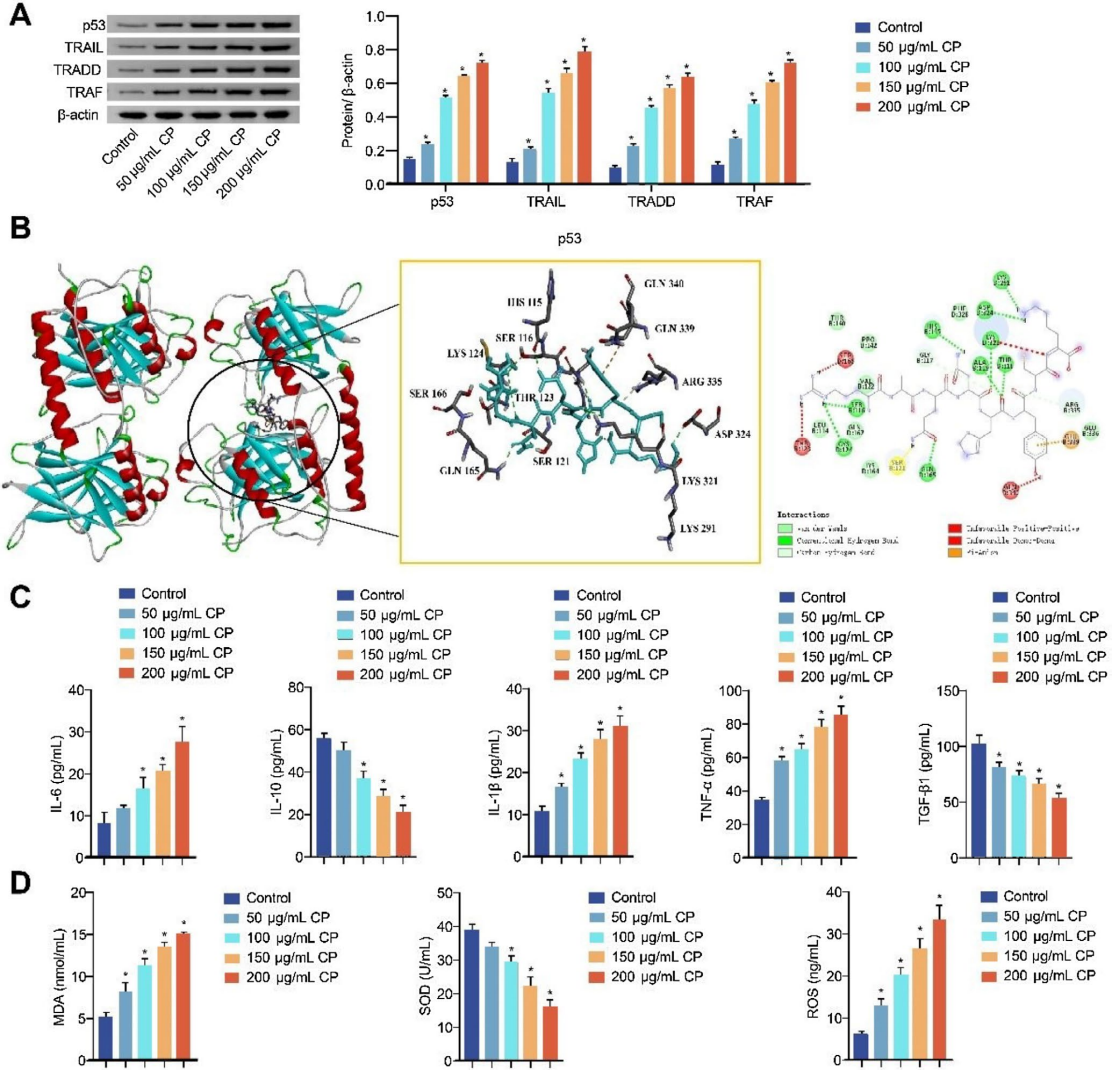
CP affected the inflammatory reaction of liver cancer cells by regulating the p53/TRAIL pathway. (A) Western blot analysed the expressions of p53, TRAIL, TRADD and TRAF in HepG2 cells; (B) Molecular docking verified the binding of CP and p53 (binding energy: −6.1 kcal/mol); (C) The levels of IL‐6, IL‐10, IL‐1β, TNF‐α and TGF‐β1 in HepG2 cell supernatant were determined by ELISA; (D) The levels of MDA, SOD and ROS in HepG2 cell supernatant were assessed by biochemical kits. The experiment was repeated three times. The data were presented as the mean ± SD. *p < 0.05 vs. control.

To further explain the effect of CP on the inflammatory reaction, we detected the expressions of p53/TRAIL pathway‐related proteins, inflammatory cytokines and markers of oxidative stress. The expressions of p53, TRAIL, TRADD and TRAF were upregulated in HepG2 cells after CP treatment (Figure 3A). Molecular docking results showed that CP bound to p53 with the binding energy being −6.1 kcal/mol. Van der Waals, conventional hydrogen bond, and carbon–hydrogen bond were the main interaction forces (Figure 3B). In addition, the levels of IL‐6, IL‐1β, TNF‐α, MDA and ROS were elevated, while those of IL‐10, TGF‐β1 and SOD were decreased in HepG2 cell supernatant after CP treatment (Figure 3C,D). These results suggest that CP may affect the inflammatory reaction of liver cancer cells, possibly via the p53/TRAIL pathway.

### 3.3 CP Affected Ferroptosis of Liver Cancer Cells by Regulating the p53/TRAIL Pathway

**FIGURE 4:**
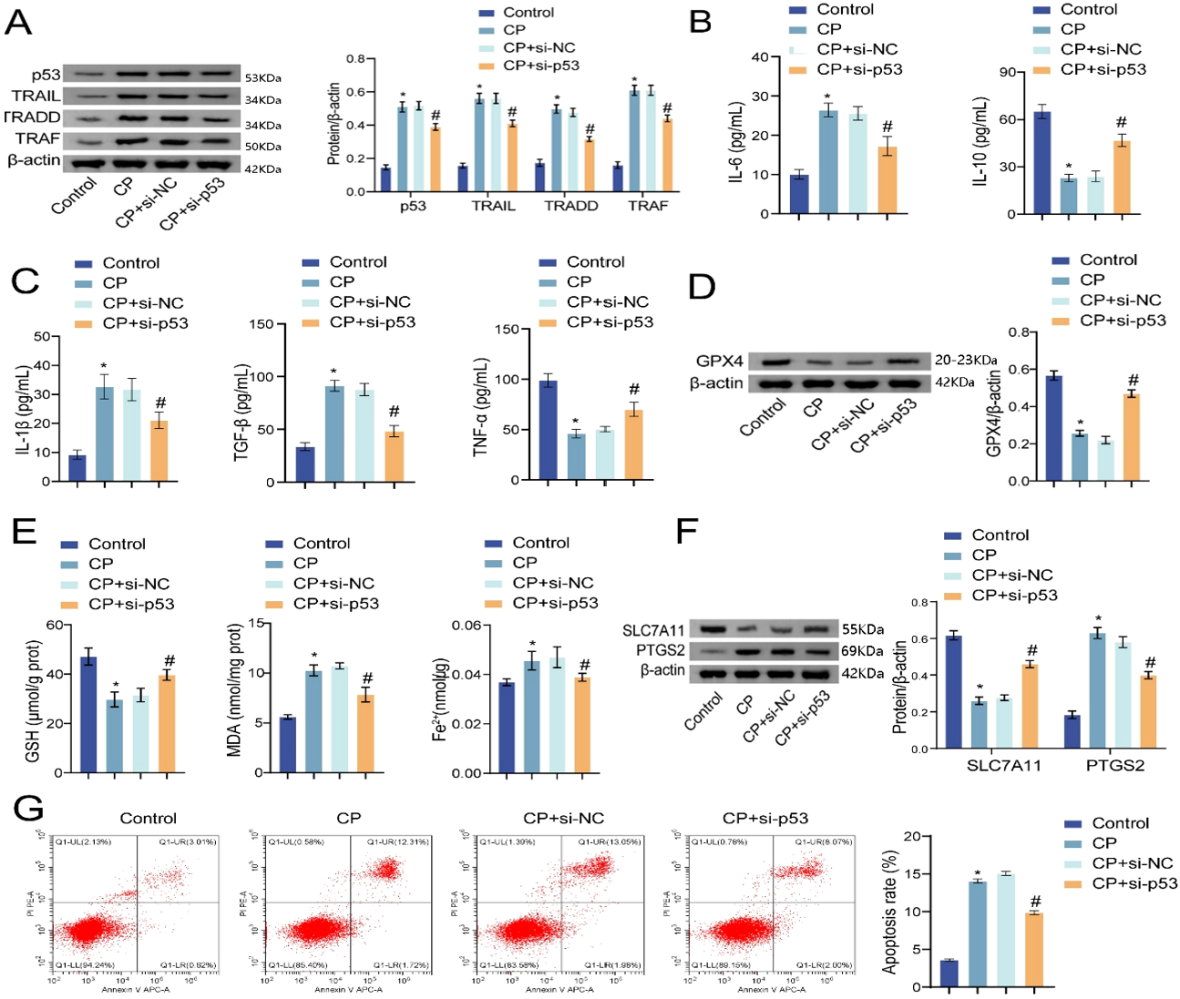
CP affected the ferroptosis of liver cancer cells by regulating the p53/TRAIL pathway. (A) Western blot analysis of the expressions of p53, TRAIL, TRADD and TRAF in HepG2 cells; (B) The enrichment of GPX4 in HepG2 cells was assessed by Western blot; (C) The levels of GSH, MDA and Fe^2+^ were measured by biochemical kits; (D) The expressions of SLC7A11 and PTGS2 in HepG2 cells were analysed by Western blot; (E) Flow cytometry detection of the apoptosis rate of HepG2 cells. The experiment was repeated three times. The data were presented as the mean ± SD. *p < 0.05 vs. control, ^#^ *p* < 0.05 vs. CP+si‐NC.

To investigate the role of the p53/TRAIL pathway in CP‐induced ferroptosis, HepG2 cells were treated with CP, CP+si‐p53 or CP+si‐NC, with untreated cells as the control group. The expression of proteins related to the p53/TRAIL pathway and ferroptosis markers was then evaluated. Compared with the control group, CP treatment upregulated the expression of p53, TRAIL, TRADD and TRAF in HepG2 cells. When treated with CP+si‐NC, the expression of these proteins showed no significant change compared with the CP group. However, treatment with CP+si‐p53 resulted in a downregulation of p53, TRAIL, TRADD and TRAF compared with the CP group (Figure 4A). Moreover, CP treatment reduced GPX4 accumulation in HepG2 cells, while cotreatment with CP and si‐p53 increased GPX4 accumulation (Figure 4B). In addition, CP treatment decreased GSH levels, increased Fe^2+^ levels and elevated MDA levels in the cell culture supernatant. These effects were reversed by cotreatment with CP and si‐p53, which led to increased GSH levels and decreased MDA and Fe^2+^ levels (Figure 4C). Further analysis showed that CP treatment downregulated SLC7A11 expression and upregulated PTGS2 expression in HepG2 cells, whereas cotreatment with CP and si‐p53 resulted in upregulated SLC7A11 and downregulated PTGS2 expression (Figure 4D). CP treatment also increased the apoptosis rate of HepG2 cells, which was reduced upon cotreatment with CP and si‐p53 (Figure 4E). The above results indicate that CP might influence the ferroptosis of liver cancer cells, potentially through the p53/TRAIL pathway.

### 3.4 CP Treated Liver Cancer

**FIGURE 5:**
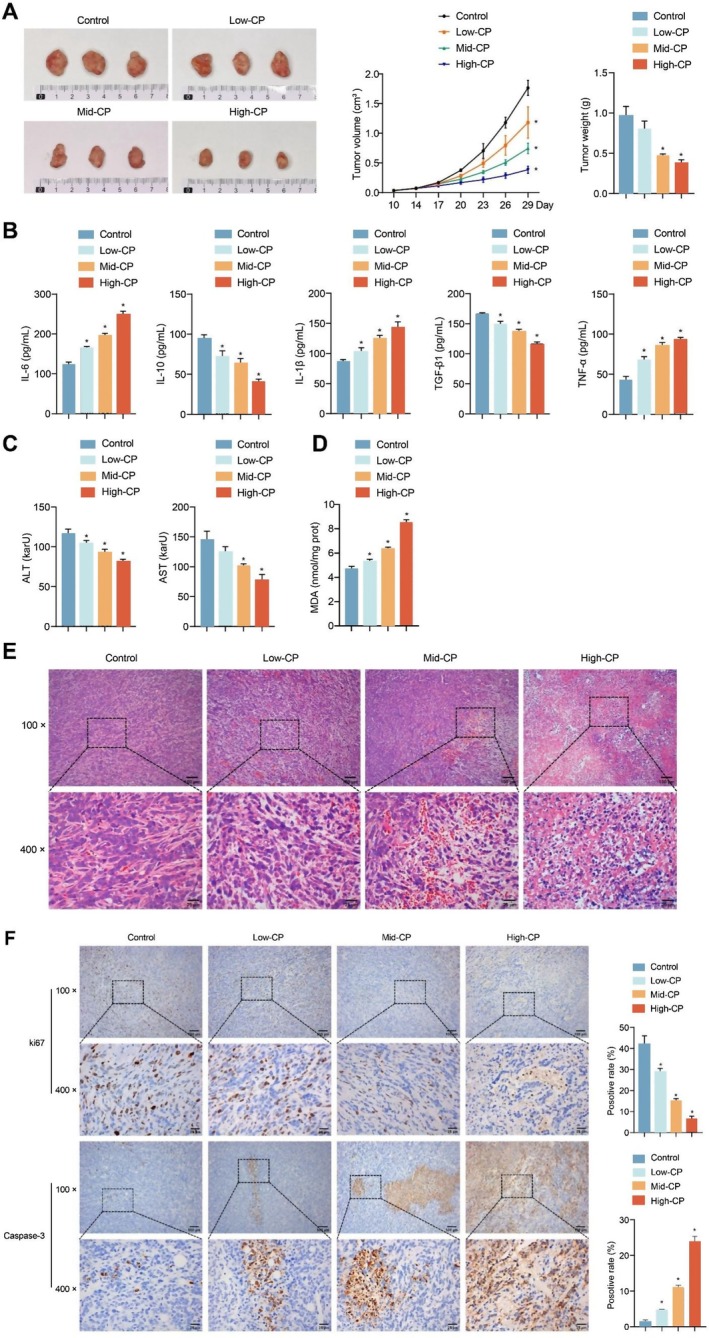
CP treated liver cancer. (A) The formation, volume and weight of tumour in nude mice; (B) The levels of IL‐6, IL‐10, IL‐1β, TNF‐α and TGF‐β1 in serum were determined by ELISA; (C) The concentrations of ALT and AST in serum were assessed by biochemical kits; (D) The levels of MDA in tumour tissues were measured by biochemical kits; (E) The histopathological changes of tumour tissues were observed by HE staining. CP treatment disrupted the structure of HepG2 cells and caused nuclear pyknosis, deepening staining and apoptosis. In addition, the damage of CP to HepG2 cells is concentration‐dependent. Scale bar: 100 μm (100×, up), 25 μm (400×, down); (F) The expressions of Ki‐67 and caspase‐3 in tumour tissues were assessed by IHC staining. Scale bar: 100 μm (100×, up), 25 μm (400×, down). *n* = 6 mice/group. The experiment was repeated three times. The data were presented as the mean ± SD. *p < 0.05 vs. control.

To expose the therapeutic effect of CP on liver cancer in vivo, the nude mouse tumourigenicity assay was conducted, and different concentrations of CP were injected intraperitoneally. The volume and weight of tumours were diminished after CP treatment (Figure 5A). The levels of IL‐6, IL‐1β and TNF‐α were raised while those of IL‐10 and TGF‐β1 were reduced in serum after CP treatment (Figure 5B). Further detection displayed that concentrations of ALT and AST in serum were decreased after CP treatment (Figure 5C). Moreover, MDA levels in tumour tissues were raised after CP treatment (Figure 5D). HE staining results showed that normal HepG2 cells were neatly arranged with complete cell structure. After CP treatment, the structure of HepG2 cells was damaged, nuclear staining was deepened and apoptosis occurred, and the changes were most obvious in the high‐CP group (Figure 5E). IHC staining further demonstrated that CP treatment reduced Ki67 expression and increased caspase‐3 expression in tumour tissues. Results for the positive and negative controls are shown in Figure S1. These proved that CP had a certain inhibitory effect on the development of liver cancer.

### 3.5 CP Affected the Inflammatory Reaction and Ferroptosis of Liver Cancer Cells Through the p53/TRAIL Pathway

**FIGURE 6:**
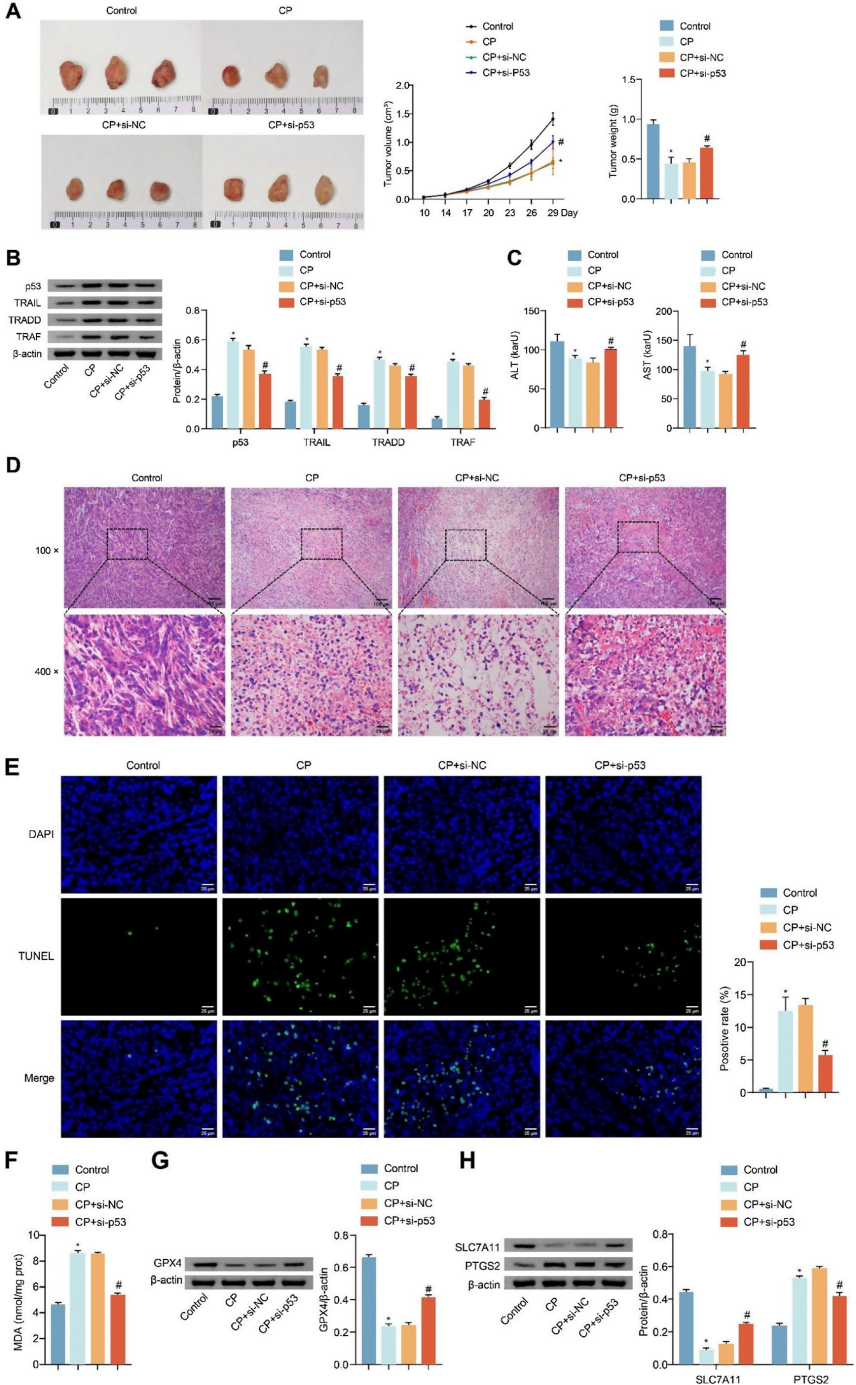
CP affected the inflammatory reaction and ferroptosis of liver cancer cells through the p53/TRAIL pathway. (A) The formation, volume and weight of tumour in nude mice; (B) Western blot analysed the expressions of p53, TRAIL, TRADD and TRAF in tumour tissues; (C) The levels of ALT and AST in serum were assessed by biochemical kits; (D) The histopathological changes of tumour tissues were observed by HE staining. CP treatment disrupted the structure of HepG2 cells and caused nuclear pyknosis, deepening staining and apoptosis. However, si‐p53 transfection reversed CP‐induced damage to HepG2 cells. Scale bar: 100 μm (100×, up), 25 μm (400×, down); (E) TUNEL staining was applied to assay the apoptosis rate of HepG2 cells in tumour tissues. Scale bar: 25 μm; (F) The levels of MDA in tumour tissues were measured by biochemical kits; (G) The enrichment of GPX4 in tumour tissues was measured by Western blot; (H) The expressions of SLC7A11 and PTGS2 in tumour tissues were analysed by Western blot. *n* = 6 mice/group. The experiment was repeated three times. The data were presented as the mean ± SD. *p < 0.05 vs. Control, ^#^ *p* < 0.05 vs. CP+si‐NC.

To verify the specific mechanism of CP in treating liver cancer in vivo, we injected si‐p53‐transfected HepG2 cells into nude mice, intervened by intraperitoneal injection of CP, and detected the level of markers related to inflammatory reaction and ferroptosis. The volume and weight of tumours were reduced after CP treatment. Compared with the CP+si‐NC group, the tumours in the CP+si‐p53 group were bigger and heavier (Figure 6A). The expression of p53, TRAIL, TRADD and TRAF was upregulated in the tumour tissues after CP treatment. Compared with the CP+si‐NC group, the expressions of p53, TRAIL, TRADD and TRAF decreased in the tumour tissues in the CP+si‐p53 group (Figure 6B). Further detection showed that the concentrations of ALT and AST in serum were decreased after CP treatment. Compared with the CP+si‐NC group, the concentrations of ALT and AST in serum in the CP+si‐p53 group were increased (Figure 6C). HE staining results showed that the HepG2 cell structure was damaged, nuclear staining was deepened and apoptosis occurred in tumour tissues after CP treatment. Compared with the CP+si‐NC group, HepG2 cells in the CP+si‐p53 group had a more complete structure and reduced apoptosis in tumour tissues (Figure 6D). TUNEL staining further revealed that CP treatment promoted the apoptosis of HepG2 cells in tumour tissues. Compared with the CP+si‐NC group, the apoptosis of HepG2 cells in tumour tissues in the CP+si‐p53 group was reduced (Figure 6E). After CP treatment, MDA levels in tumour tissues rose. Compared with the CP+si‐NC group, MDA levels in tumour tissues declined in the CP+si‐p53 group (Figure 6F). Western blot further verified that the expressions of GPX4 and SLC7A11 were downregulated, and that of PTGS2 was upregulated in tumour tissues after CP treatment. Compared with the CP+si‐NC group, the expressions of GPX4 and SLC7A11 were upregulated, and that of PTGS2 was downregulated in tumour tissues in the CP+si‐p53 group (Figure 6G,H). These results displayed that p53 plays a role in liver cancer and may exert pro‐tumorigenic effects. CP can modulate p53 and influence inflammatory responses and ferroptosis in liver cancer cells through the p53/TRAIL pathway. (Note: each group included only three biological replicates for in vivo experiments, which may limit the statistical robustness.)

## 4 Discussion

CP extracted from *Scolopendra subspinipes* mutilans [15], has demonstrated inhibitory effects on multiple liver cancer cell lines in vitro, primarily through the modulation of ferroptosis and inflammatory responses. These findings were further substantiated by in vivo experiments, which confirmed the antitumour efficacy of CP. The results suggest that CP may exert its effects via activation of the p53/TRAIL signalling pathway, although further validation is required to confirm this mechanism.

TCM has long recognised the medicinal value of centipede and its extracts in tumour suppression, especially for liver cancer [14, 15]. Herbal compounds such as luteolin and artemisinin derivatives have been shown to induce apoptosis in hepatocellular carcinoma (HCC) cells by upregulating TRAIL receptors DR4 and DR5 [42, 43]. Additionally, other studies have reported the application of centipede extracts in treating soft tissue sarcoma and EGFR‐dependent malignancies [12, 13]. These observations support the hypothesis that CP may influence HCC progression through p53/TRAIL‐mediated signalling.

Previous studies have characterised the structure and anticancer potential of CP, identifying its molecular formula (C42H66N16O12S) and its ability to activate TRAIL receptors. However, the regulatory role of upstream molecules such as p53 had not been explored. As a critical transcription factor, p53 is activated in response to DNA damage and cellular stress and is known to initiate apoptosis by regulating TRAIL expression [30, 44]. Furthermore, prior work has largely focused on apoptosis, overlooking the potential involvement of ferroptosis and inflammatory mechanisms.

His study further builds upon previous findings by systematically investigating the role of CP in regulating the tumour inflammatory microenvironment and ferroptosis. Inflammation is closely associated with the progression of HCC [45]. Our results showed that CP significantly upregulated IL‐6, IL‐1β and TNF‐α while downregulating IL‐10 and TGF‐β1, indicating its pro‐inflammatory activity. Oxidative stress is a critical mechanism contributing to liver injury, leading to excessive accumulation of MDA and ROS [46]. CP treatment elevated MDA and ROS levels and reduced SOD expression, suggesting that it may exert anticancer effects by aggravating oxidative damage. Ferroptosis plays a pivotal role in the drug‐induced death of HCC cells [47]. In this regard, CP downregulated SLC7A11, GPX4 and GSH levels while increasing Fe^2+^ and PTGS2 expression, supporting its role in promoting ferroptosis.

Molecular docking analysis revealed that CP forms a stable complex with p53 through hydrogen bonding and van der Waals interactions. At both cellular and tumour tissue levels, CP significantly upregulated the expression of p53, TRAIL, TRADD and TRAF, suggesting activation of the p53/TRAIL signalling pathway. Moreover, CP enhanced caspase‐3 expression while reducing Ki67 levels, indicating its ability to induce apoptosis and inhibit tumour cell proliferation [48, 49]. Notably, si‐p53 transfection reversed the antitumour effects of CP, further confirming the critical role of p53 in mediating this process.

In summary, this study reveals that CP suppresses liver cancer progression by promoting ferroptosis and inflammatory responses, potentially through the p53/TRAIL signalling pathway. These findings provide a theoretical basis for the clinical application of CP in liver cancer therapy. Although this study provides a theoretical basis for the clinical application of CP, several limitations remain: the direct regulatory effect of p53 on TRAIL requires further validation; the sample size of in vivo experiments was relatively small; and the long‐term toxicity and pharmacokinetic profile of CP have not yet been evaluated. Moreover, the development and progression of hepatocellular carcinoma may involve many other complex molecular pathways and mechanisms. Future research will focus on optimising the delivery system of CP, exploring its interactions with other potential molecular targets to better understand its anticancer effects, and investigating its synergistic effects with other anticancer agents.

## 5 Conclusions

This study further confirmed the therapeutic effect of CP on liver cancer and revealed that it may be achieved by modulating inflammatory responses and ferroptosis in liver cancer cells via the p53/TRAIL pathway. This study will provide new insights into the development of liver cancer treatment and the clinical application of CP.

## Author Contributions

**Xingru Xing:** conceptualization (equal). **Linzhu Lu:** conceptualization (equal). **Zhen Huang:** conceptualization (equal). **Jiawei Wang:** conceptualization (equal). **Lihuai Wang:** conceptualization (equal). **Yuxing Hu:** conceptualization (equal). **Shan Yin:** conceptualization (equal). **Sha Tian:** conceptualization (equal).

## Ethics Statement

The animal experiment was approved by the Hunan University of Chinese Medicine Committee (No. LLBH‐202206240002).

## Conflicts of Interest

The authors declare no conflicts of interest.

## Supporting information


**Figure S1:** Positive and negative controls for the expression of Ki‐67 and caspase‐3 by IHC staining. Scale bar: 100 μm (100×, up), 25 μm (400×, down). The blue colour represents the nucleus stained with haematoxylin. The brown colour represents the precipitate after the reaction of diaminobenzidine (DAB) and horseradish peroxidase (HRP), representing the positive expressions of Ki‐67 and caspase‐3.

## Data Availability

All data were presented in the text, and the original data can be obtained from the corresponding author upon request.
