# Supplementary material for: Centipede Polypeptide Affects the Inflammatory Reaction and Ferroptosis of Liver Cancer Cells Through the p53/TRAIL Pathway
Source: J Cell Mol Med. 2025 Oct 13;29(19):e70844. doi: 10.1111/jcmm.70844 (PMC12516244; doi:10.1111/jcmm.70844)
Supplement: Supplementary file 1 — Figure S1: Positive and negative controls for the expression of Ki‐67 and caspase‐3 by IHC staining. Scale bar: 100 μm (100×, up), 25 μm (400×, down). The blue colour represents the nucleus stained with haematoxylin. The brown colour represents the precipitate after the reaction of diaminobenzidine (DAB) and horseradish peroxidase (HRP), representing the positive expressions of Ki‐67 and caspase‐3. [file JCMM-29-e70844-s001.docx]

**Supplementary Material**

**
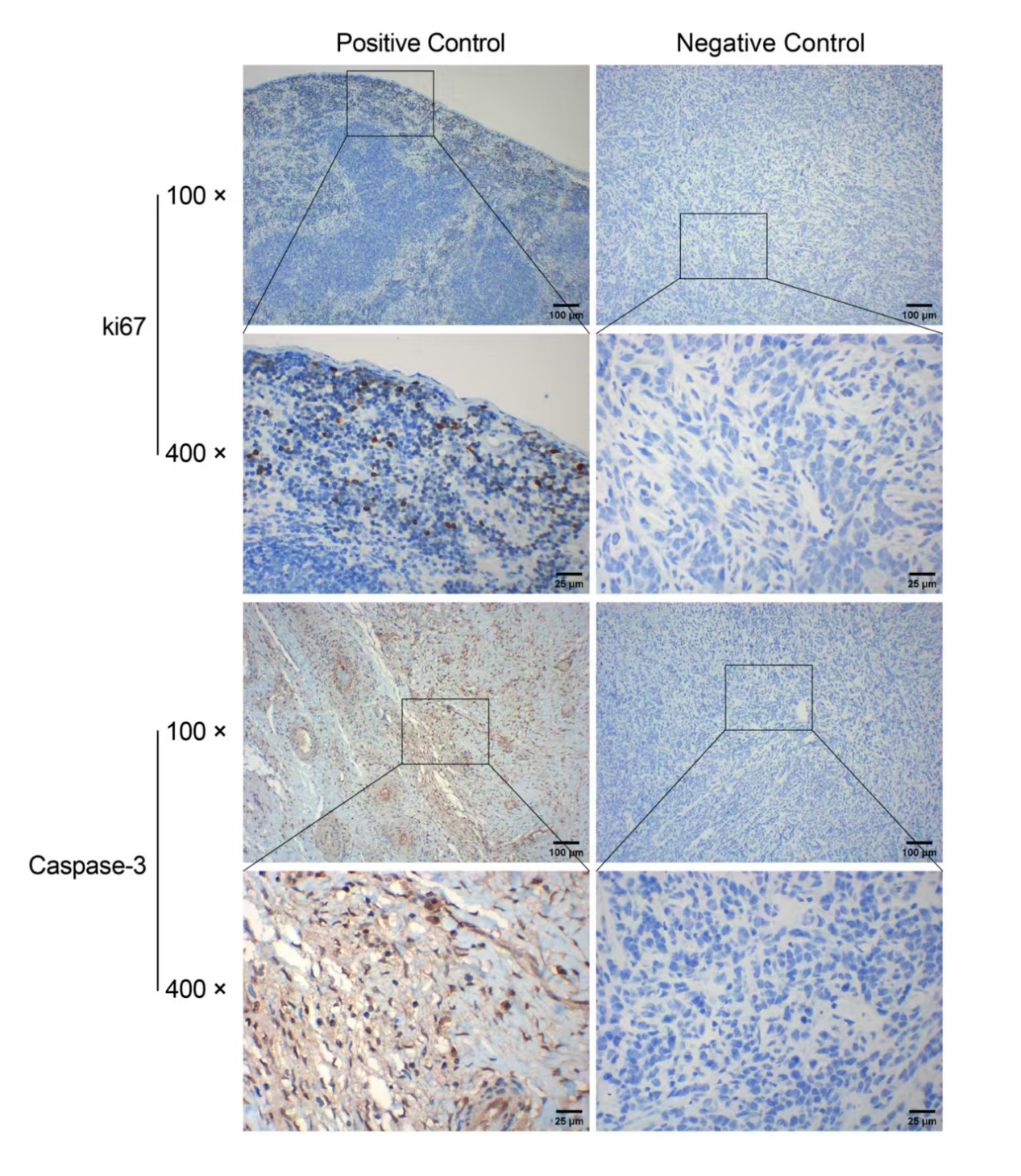
**

**Figure S1. Positive and negative controls for the expression of Ki-67 and Caspase-3 by IHC staining.** Scale bar: 100 μm (100×, up), 25 μm (400×, down). The blue color represents the nucleus stained with hematoxylin. The brown color represents the precipitate after the reaction of diaminobenzidine (DAB) and horseradish peroxidase (HRP), representing the positive expressions of Ki-67 and Caspase-3.
